# Draft Genome Sequence of *Deinococcus indicus* DR1, a Novel Strain Isolated from a Freshwater Wetland

**DOI:** 10.1128/genomeA.00754-17

**Published:** 2017-08-03

**Authors:** Deepika Chauhan, Pulkit Anupam Srivastava, Ragothaman M. Yennamalli, Richa Priyadarshini

**Affiliations:** a Department of Life Sciences, School of Natural Sciences, Shiv Nadar University, Gautam Buddha Nagar, Uttar Pradesh, India; b Department of Biotechnology and Bioinformatics, Jaypee University of Information Technology, Waknaghat, Himachal Pradesh, India

## Abstract

*Deinococcus indicus* strain DR1, a red-pigmented, arsenic- and radiation-resistant bacterium, was isolated from a water sample of the Dadri wetland, Uttar Pradesh, India. Here, we report a draft genome sequence of this strain, which may provide useful information regarding the genes and pathways involved in heavy-metal bioremediation.

## GENOME ANNOUNCEMENT

The *Deinococcus* genus is known for its extreme resistance to UV and gamma-ray radiation (1, 2). Members of this genus are aerobic non-spore-forming chemoorganotrophs and produce the carotenoid pigment deinoxanthin that gives them their characteristic pink color. Although most of the species are Gram-positive and spherical in shape, a few species are Gram-negative and rod-shaped. We isolated the light-pinkish, arsenic- and radiation-resistant *Deinococcus indicus* strain DR1 from Dadri wetland, Uttar Pradesh, India. This bacterium was found to be Gram-negative and rod-shaped.

Genomic DNA was extracted by a conventional isolation method described previously (3). The whole genome of *Deinococcus indicus* strain DR1 was sequenced using the HiSeq 2500 sequencing platform (Illumina), with a paired-end module, and was performed at AgriGenom Labs Pvt Ltd., Cochin, India. A total of 7,965,538 paired-end reads were collected using Illumina HiSeq 2500. Cutadapt version 1.8.1 (4) was used for adapter trimming of Illumina reads, followed by low-quality data filtration using Sickle version 1.33 (https://github.com/najoshi/sickle). To avoid the serious impact of duplicates found in whole-genome sequencing on contig generation, scaffolding, and discovery of genome variations, Fastuniq-1.1 was used to discard duplicates (5). This filtration resulted in 40 contigs, with a GC content of 69% and sizes ranging from 989,508 to 10,905 bp (mean length, 111,690.7 bp), containing 4,467,628 bp in total. CISA was used to perform the final *de novo* assembly of contigs (6). Annotation of the assembled genome was performed by the NCBI Prokaryotic Genome Annotation Pipeline (PGAP).

The draft genome contains a total of 4,218 genes, including 4,151 coding sequences (CDSs) and 67 RNA genes. In addition, complete 5S rRNA and partial 6S rRNA and 23S rRNA gene sequences were also annotated. As *D. indicus* is a rod-shaped bacterium, genes involved in maintaining the rod shape, such as those encoding rod shape-determining protein MreB, rod shape-determining protein MreC, and rod shape-determining protein RodA, were identified.

We mined the genome for the presence of heavy-metal resistance genes, since *D. indicus* can grow in the presence of heavy metals, such as arsenic (7). Using BLAST analysis, we found the presence of genes coding for an arsenic efflux membrane protein (*arsB*), a trancriptional regulator (*arsR*), and an arsenate reductase (8). Genes involved in mercury (9), copper, and chromate resistance were also identified (10, 11). Other heavy-metal-associated genes, such as heavy-metal-translocating P-type ATPase, heavy-metal transport/detoxification protein, and heavy-metal-associated domain-containing protein, were also identified in this genome. Further physiological studies are being performed for the bioremediation potential of *D. indicus* strain DR1.

### Accession number(s).

This whole-genome shotgun project has been deposited at DDBJ/ENA/GenBank under the accession no. NHMK00000000. The version described in this paper is version NHMK01000000.
